# A common monitoring framework for ending preventable maternal mortality, 2015–2030: phase I of a multi-step process

**DOI:** 10.1186/s12884-016-1035-4

**Published:** 2016-08-26

**Authors:** Allisyn C. Moran, R. Rima Jolivet, Doris Chou, Sarah L. Dalglish, Kathleen Hill, Kate Ramsey, Barbara Rawlins, Lale Say

**Affiliations:** 1 US Agency for International Development, Global Health Bureau, Washington, DC USA; 2 Maternal Health Task Force, Boston, MA USA; 3 World Health Organization, Geneva, Switzerland; 4 Maternal and Child Survival Program, Jhpiego, Washington, DC USA; 5 Averting Maternal Death and Disability Program, Columbia University, New York, NY USA

**Keywords:** Maternal mortality, Indicators, Monitoring and evaluation

## Abstract

**Background:**

While global maternal mortality declined 44 % between 1990 and 2015, the majority of countries fell short of attaining Millennium Development Goal targets. The Sustainable Development Goals (SDGs), adopted in late 2015, include a target to reduce national maternal mortality ratios (MMR) to achieve a global average of 70 per 100,000 live births by 2030. A comprehensive paper outlining *Strategies toward Ending Preventable Maternal Mortality (EPMM)* was launched in February 2015 to support achievement of the SDG global targets. To date, there has not been consensus on a set of core metrics to track progress toward the overall global maternal mortality target, which has made it difficult to systematically monitor maternal health status and programs over time.

**Findings:**

The World Health Organization (WHO), Maternal Health Taskforce (MHTF), and the US Agency for International Development (USAID) along with its flagship Maternal and Child Survival Program (MCSP), facilitated a consultative process to seek consensus on maternal health indicators for global monitoring and reporting by all countries. Consensus was reached on 12 indicators and four priority areas for further indicator development and testing. These indicators are being harmonized with the Every Newborn Action Plan core metrics for a joint global maternal newborn monitoring framework. Next steps include a similar process to agree upon indicators to monitor social, political and economic determinants of maternal health and survival highlighted in the EPMM strategies.

**Conclusion:**

This process provides a foundation for the maternal health community to work collaboratively to track progress on core global indicators. It is important that actors continue to work together through transparent and participatory processes to track progress to end preventable maternal mortality and achieve the SDG maternal mortality targets.

**Electronic supplementary material:**

The online version of this article (doi:10.1186/s12884-016-1035-4) contains supplementary material, which is available to authorized users.

## Background

In September 2015, the era of the Millennium Development Goals (MDGs) came to an end, and while the worldwide maternal mortality ratio (MMR) declined by 44 % from 1990 to 2015, the global target of a 75 % reduction was not reached. In fact, the majority of countries fell short of attaining MDG5 targets for maternal mortality reduction. Maternal mortality remains unacceptably high with approximately 303,000 maternal deaths occurring each year, with the largest burden in Sub-Saharan Africa and Asia [1].

Beginning in January 2013, the Ending Preventable Maternal Mortality (EPMM) Working Group, led by the World Health Organization (WHO) with support from partner organizations, achieved multi-stakeholder consensus on goals for maternal health and survival from 2015 to 2030.

The EPMM targets for maternal mortality reduction at the global and country levels are:

- By 2030, the global average maternal mortality ratio (MMR) should be less than 70 maternal deaths per 100,000 live births
- By 2030, every country should reduce its maternal mortality ratio by at least two thirds from 2010 baseline, and no country should have a ratio higher than 140 deaths per 100,000 live births (twice the global target)
- All countries are tasked with achieving equity in MMR among sub-populations

A comprehensive paper outlining the *Strategies toward Ending Preventable Maternal Mortality (EPMM)* was released by WHO in February 2015 [2]. This was followed by a synthesis of priorities for ending preventable maternal and newborn deaths and stillbirths in a series of technical papers in support of the updated United Nations Global Strategy for Women’s, Children’s, and Adolescent’s Health (2016–2030) [3].

The EPMM global MMR target was incorporated into the Sustainable Development Goals (SDGs) adopted by member states and launched in late 2015 [4]. The SDGs form the basis of a new global development agenda, which is broad and comprehensive, covering a wide range of social, economic and environmental goals. The EPMM global target was also included in the updated UN Global Strategy.

To achieve the ambitious SDG and Global Strategy MMR targets, it will be imperative to accelerate coverage of quality essential services and to address the underlying social, political and economic determinants of maternal health across all settings. To support implementation of EPMM strategies and to track progress toward global and country MMR targets, consensus on priority, methodologically robust maternal health (MH) indicators is urgently needed. To this end, in May 2015, the EPMM Working Group specified a plan to develop a comprehensive maternal health global monitoring and reporting framework in two phases:

1. Phase 1: Consensus on a core set of priority, methodologically robust maternal health indicators with direct relevance for reducing preventable mortality (proximal to causes of death) for global monitoring and reporting by all countries
2. Phase 2: Consensus on complementary indicators to track progress toward addressing the social, political and economic determinants of maternal health and survival (distal to causes of death) highlighted in the global EPMM strategy for testing by countries as part of national health plans and monitoring frameworks

This paper describes the process and results of Phase I – to develop a core set of maternal health indicators for global monitoring and reporting by all countries. This core set of maternal health indicators will be harmonized with Every Newborn Action Plan (ENAP) core metrics to create a joint global maternal and newborn monitoring framework [5].

## Methods

**Fig. 1:**
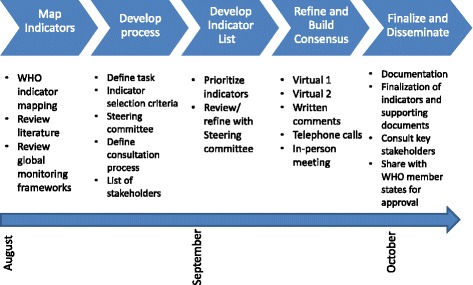
Process for Consensus Building, August-October, 2015

The World Health Organization, Maternal Health Taskforce (MHTF), and the US Agency for International Development (USAID) along with its flagship Maternal and Child Survival Program (MCSP), facilitated an iterative consultative process to reach consensus on a core set of maternal health indicators for global monitoring and reporting under Phase I on behalf of the EPMM Working Group. Key phases of the process led by an EPMM-designated steering committee included: (1) structured mapping exercise to identify indicators for monitoring and evaluation of maternal health; (2) development of key principles, criteria, and consultative process for selection of EPMM phase I indicators; (3) selection of experts/stakeholders to participate in consultative process in line with agreed criteria; (4) facilitation of a series of virtual consultations via webinar, phone and in writing to rank and prioritize indicators based on a set of pre-determined questions and criteria; and (5) an in-person one-day technical meeting with identified experts/stakeholders to reach consensus on a core set of MH indicators for global monitoring (Fig. 1).

The EPMM-designated steering committee included six persons selected to represent a range of expertise, including technical, policy and measurement experts, who were tasked with overseeing the process of defining a core set of global maternal health indicators (Table 1). The Steering Committee identified four principles to guide development and prioritization of a set of core MH indicators for global monitoring and reporting. First, the scope was limited to priority indicators appropriate for global monitoring and reporting by all countries. It was agreed that the primary purpose of the indicators would be to track progress toward maternal mortality reduction at the global level, with no more than 10 to 12 indicators in total. It was acknowledged that complementary (and sometimes overlapping) indicators would be needed to support effective program management at sub-national and service delivery levels but that these indicators were beyond the scope of the EPMM global indicator exercise. During the process, indicators for other levels of monitoring and reporting were systematically captured and recorded for future research and development, building on numerous ongoing indicator development efforts. A second guiding principle was to limit Phase I indicators to measures directly linked to the proximal causes of maternal death to mirror the core set of newborn health metrics outlined in the Every Newborn Action Plan. More distal determinants, such as social, economic, political, and health systems factors, will be developed under Phase II of the EPMM process.

**Table 1:** Steering Committee Members^a^

Name	Organization	Job title	Training, area of expertise
Allisyn Moran, PhD MHS	US Agency for International Development	Senior Maternal Health Advisor	Monitoring and evaluation, research, maternal and child health
R. Rima Jolivet, CNM, DrPH	Maternal Health Task Force	Maternal Health Technical Director	Certified nurse-midwife, public health, maternal health system strengthening, quality improvement
Kathleen Hill, MD	Maternal and Child Survival Program, Jhpiego	Team Lead, Maternal Health	Family physician, public health, service delivery, program implementation, quality improvement
Barbara Rawlins, MPH	Maternal and Child Survival Program, Jhpiego	Team Lead, Monitoring and Evaluation	Monitoring and evaluation, research, maternal and child health
Lale Say, MD	World Health Organization, Department of Reproductive Health and Research	Coordinator, adolescents and at-risk populations Reproductive Health and Research	Physician, monitoring and evaluation, research, program strengthening
Sarah Dalglish, PhD	World Health Organization, Department of Reproductive Health and Research	Consultant	Political Economy, International Relations, Health Politics and Policy
Kate Ramsey, MPH, DrPH (c) (position at the time of this project)	Averting Maternal Death and Disability Program, University of Columbia	Senior Research Officer	Public health, maternal and newborn health service delivery, health systems, implementation research

^a^No financial conflicts declared by any members of the Steering Committee

The third agreed principle was to use the same organizational categories of these ENAP metrics, to facilitate a harmonized global maternal and newborn monitoring framework. Thus it was agreed to retain the ENAP metrics organizational categories of impact, coverage and input indicators. Finally, it was assumed that indicators would be reported at least every 5 years to track progress toward global targets.

In parallel, the steering committee finalized indicator selection criteria, drawing from criteria used in other indicator prioritization activities (e.g. WHO 100 core indicators [6]). Selection criteria included: (1) relevance for directly reducing preventable mortality; (2) validity (measures what it is supposed to measure); (3) feasibility (feasible to measure routinely through household surveys, health facility assessments, routine information systems); (4) degree of data availability across countries; and (5) strengthens or complements existing monitoring frameworks (MDGs, SDGs; ENAP; and other global efforts (e.g. Global Strategy for Women's, Children's, and Adolescent's Health; WHO Quality of Care Initiative, etc.)). The WHO indicator mapping exercise (phase one above) included searching online databases, reviewing program documents and other global monitoring frameworks, and discussions with implementing partners and donor agencies. An initial expert consultation was conducted in May 2015. Applying these principles and selection criteria, the Steering Committee compiled the indicators prioritized during this consultation, as well as priority indicators for tracking SDGs, and other indicator mapping efforts. This list was refined and updated, which resulted in a final list of 20 to 25 indicators.

In the second phase a series of virtual consultations was conducted with selected expert participants. Key stakeholders and experts were identified through review of participant lists from other indicator/measurement consultations, discussions with the broader EPMM Working Group, and other relevant stakeholders. The focus was on identifying participants who work directly in measurement of maternal health including clinicians, researchers, program implementers, global policy makers and representatives from Ministries of Health, UN agencies, and donors. (Table 2). Based on the structured indicator review and input from measurement experts, the Steering Committee developed key questions related to the underlying principles and indicator selection criteria to guide discussion and feedback during a series of virtual consultations. In each virtual technical review session, the indicators were discussed, guided by the key questions. Feedback from previous sessions was also shared, so learnings were incorporated and refined during successive phases. Written feedback was solicited from those unable to participate in planned consultations.

**Table 2:** List of participant organizations

Organization	Department, Location	Specialty	Phase of process						
			EPMM core group	Steering committee	Virtual 1	Virtual 2	In-person meeting	Written comments	Invited, unable to participate
UN agencies									
UNFPA	MN Technical Division	Programs, M&E, Clinical	X		X	X	X		
UNICEF	Division of Data, Research and Policy	Epidemiology, Research							
World Health Organization	MCA, RHR	Epidemiology, M&E, Clinical	X	X	X	X	X		
Programs									
Averting Maternal Death and Disability Program (AMDD)^a^	Columbia University SPH	Research, Programs, M&E		X	X	X	X		
Family Care International^b^		Advocacy, Programs				X			X
ICF Macro^c^	Demographic and Health Surveys (DHS)	MH Measurement					X		
Improving Coverage Measurement^d^	JHU, Institute for International Programs	Epidemiology, Research					X	X	
Maternal and Child Survival Program^e^	Jhpiego	M&E, Research, Program, Policy, Clinical	X	X	X	X	X		
Maternal Health Task Force^f^	Harvard, SPH	, Advocacy, Clinical	X	X	X	X	X		
MEASURE Evaluation^g^	University of North Carolina, Chapel Hill	M&E, Programs			X		X		
Population Council^h^		Evaluation and Research, Programs					X		
White Ribbon Alliance^i^		Advocacy					X		
Universities, Research Institutes									
icddr,b	Dhaka, Bangladesh	Research, Surveillance, Programs, Clinical				X	X		
Ifakara Health Institute	Dar es Salaam, Tanzania	Research, Surveillance, Programs, Clinical					X		
Johns Hopkins University	Baltimore, MD, USA	Research							X
London School of Hygiene and Tropical Medicine	London, UK	Research						X	
Makerere University	Kampala, Uganda	Research, Surveillance, Programs, Clinical							X
University of Aberdeen	Scotland	Research							X
University of Heidelberg		Research Programs					X	X	
University of Ouagadougou	Ouagadougou, Burkina Faso	Research, Programs, Clinical					X		
University of Southampton	Southampton, UK	Research					X	X	
Ministries of Heatlh									
Department of Health, South Africa		Policy, Programs, M&E, Clinical					X		
Ministry of Health, Kenya		Policy, Programs, M&E, Clinical							X
Ministry of Health, Nigeria		Policy, Programs, M&E, Clinical							X
Consultants									
Ethiopia		Research, M&E					X		
Ghana		Clinical, Programs, Research					X		
Kazakhstan		Clinical, Research maternal death and response							X
Donors									
Bill & Melinda Gates Foundation	Seattle, WA USA	Clinical, M&E, Research						X	X
Children’s Investment Fund Foundation	London, UK	M&E, Research							X
US Agency for International Development	Washington, DC USA	Programs, M&E, Research, Clinical	X	X	X	X	X		

Note: The “Clinical” specialty refers to OB/GYN, general practitioners, midwives, and other medical professions; M&E refers to monitoring and evaluation

^a^AMDD: https://www.mailman.columbia.edu/research/averting-maternal-death-and-disability-amdd

^b^FCI: http://www.familycareintl.org/en/home

^c^DHS: http://www.dhsprogram.com/

^d^ICM: http://www.jhsph.edu/research/centers-and-institutes/institute-for-international-programs/current-projects/improving-coverage-measurements-for-mnch/

^e^MCSP: http://www.mcsprogram.org/

^f^MHTF: https://www.mhtf.org/

^g^MEASURE: http://www.cpc.unc.edu/measure/

^h^PopCouncil: http://www.popcouncil.org/

^i^WRA: http://whiteribbonalliance.org/

To culminate the consultation process, an in-person meeting was held in Washington, DC in mid-September 2015, bringing together a broader group of experts. The meeting was convened by the Maternal and Child Survival Program with financial support from the US Agency for International Development (USAID). Some participants were supported through funding from other institutions and development partners. This in-person consultation was guided by the same key questions addressed in previous virtual session. During the in-person meeting, participants considered the results from the first rounds of feedback to deliberate and reach consensus on a final core set of maternal health indicators for global monitoring and reporting. The in-person one-day consultation also addressed commonly perceived gaps in maternal health indicators, proposals for additional indicators and other issues raised by participants. Inputs from all steps of the consultation (virtual, written and in-person) were compiled in a matrix summarizing feedback and main discussion points (Additional file 1). A report of the in-person consultation highlighting the main discussion points was also developed.

## Results

In total, 45 experts from 11 countries participated in one or more stages of the structured consultative process to reach consensus on a core set of maternal health indicators for global monitoring and reporting. The experts agreed on 12 maternal health indicators - three impact, seven coverage, and two input indicators (Table 3). Five indicators overlap with the Every Newborn Action Plan core metrics, six indicators overlap with the WHO list of 100 core indicators, and one indicator overlaps with the WHO 2013 Quality of MNCH care indicators [5–7] (Table 3; Additional file 2). The definitions, disaggregators, and data sources to accompany the Core Maternal Health Indicators for Global Monitoring and Reporting have been finalized (Table 4). Four areas were prioritized for further research and testing, namely content of antenatal care, content of postpartum care, respectful maternity care, and Met Need for Emergency Obstetric Care (EmOC).

**Table 3:** Core Maternal Health Indicators for Global Monitoring and Reporting

	Indicator	Priority areas for indicator development
Impact	1. Maternal mortality ratio^a^ ^c^	
	2. Maternal cause of death (direct/indirect) based on ICD-MM	
	3. Adolescent birth rate^c^	
COVERAGE: care for all women and girls	4. Four or more antenatal care visits^c^	Content of antenatal care^b^
	5. Skilled attendant at birth^a^ ^c^	Content of postnatal care
	6. Institutional delivery	Respectful maternity care^b^
	7. Early postnatal/postpartum care for woman and baby (within 2 days of birth)^a^ ^c^	
	8. Met need for family planning^c^	
	9. Uterotonic immediately after birth for prevention of post-partum hemorrhage (among facility births)^b^	
COVERAGE: care for women and girls with complications	10. Caesarean section rate^a^	Met need for Emergency Obstetric Care
INPUTS: counting	11. Maternal death registration	
INPUT: Availability of care	12. Availability of functional Emergency Obstetric Care facilities^a^	

^a^ENAP indicator

^b^Link to WHO Quality of Care metrics

^c^Link to WHO 100 Core indicators

NOTES:

- WHO will propose a definition for maternal death registration

- Availability of functional emergency obstetric care facilities requires additional definition - The current definition will be used in the short term, with ongoing efforts to improve definition (both numerator and denominator)

- Countries should continue to monitor met need for emergency obstetric are and update definitions once they are finalized based on ongoing work

- Additional priority indicators – Efforts will link with ongoing efforts such as WHO Antenatal Care Guideline revision process, WHO Quality of Care Initiative, Global Strategy for Women’s, Children’s, and Adolescent Health

- Currently, antenatal and postnatal care are measured through the number of contacts with the health system (as defined as “visits”), but the quality or content of these visits are not assessed. Two of the priority areas moving forward include defining core content for these antenatal and postnatal visits as well as measures to assess those the core content areas

- Content of antenatal care could include: blood pressure, testing and treatment of infectious disease, counseling on danger signs, testing for HIV/AIDS, prevention of malaria during pregnancy, birth planning, etc. Content of postpartum care could include: monitoring bleeding, counseling for family planning, observing breastfeeding, counseling and assessment of postpartum depression, etc

**Table 4:** Core Maternal health indicators for global monitoring and reporting – definitions and data sources

	Indicator	Definition	Numerator	Denominator	Disaggregation	Data Source(s)
Impact	Maternal Mortality Ratio^a^ ^c^	Death from any cause related to or aggravated by pregnancy or its management (excluding accidental or incidental causes) during pregnancy, childbirth or within 42 days of termination of pregnancy, per 100,000 live births for a specified time period	Number of maternal deaths	Per 100,000 live births in the same period	Sub-national	HH surveys, CRVS, admin, modeled estimates. RAMOS, Confidential Inquiries, Census
	Maternal cause of death (proportion)	Deaths from any cause related or aggravated by pregnancy or its management (excluding accidental or incidental causes) during pregnancy, childbirth or within 42 days of termination of pregnancy for a specified time period (using ICD-MM)	Number of maternal deaths by cause	Total number of maternal deaths in the same period	Cause, Indirect/Direct	HH surveys, CRVS, admin, modeled estimates, RAMOS, Confidential Inquiries
	Adolescent birth rate^c^	Number of births to women 15 to 19 years of age per 1,000 women within specified time period	Number of births to women 15 to 19 years of age	Per 1,000 women 15 to 19 years of age in the same period	Sub-national	HH surveys, census, CRVS
COVERAGE: care for all women and girls	Four or more ANC visits^c^	Proportion of pregnant women and girls who have made at least four antenatal care visits within specified time period	Number of women and girls who received ANC at least four times during pregnancy	Total number of women and girls with a live birth in the same period	Wealth quintile Residence Age Sub-national	HH surveys
	Skilled attendant at birth^a^ ^c^	Proportion of births attended by skilled health personnel (doctor, nurse, midwife) within a specified time period	Number of live births attended by skilled health personnel	Total number of live births in the same period	Wealth quintile Residence Age Sub-national	HH surveys
	Institutional delivery	Proportion of births in a health facility within a specified time period	Number of live births in a health facility	Total number of live births in the same period	Wealth quintile Residence Age Sub-national	HH surveys
	Oxytocin immediately after birth for prevention of post-partum hemorrhage^b^	Proportion of women and girls who gave birth in a facility receiving oxytocin immediately after birth within a specified time period	Number of women and girls who gave birth in a facility who received oxytocin immediately after birth	Total number women and girls with a facility birth in same period	Sub-national Age	HMIS, Facility records
	Early postnatal/postpartum care for women and babies (within 2 days of birth)^a^ ^c^	Proportion of women/girls with a recent birth and their babies who received postnatal care within two days of birth (regardless of place of delivery) within a specified time period	Number of women/girls and their babies who received postnatal care within two days of childbirth	Total number of women/girls with a last live birth in the same period	Wealth quintile Residence Age Sub-national	HH surveys
	Met need for family planning^c^	Proportion of women and girls, either married or in a union, who have their need for contraception satisfied within a specified time period	Number of women and girls who have their need for contraception satisfied	Total number of women and girls, married or in union, in need of contraception in same period	Wealth quintile Residence Age Sub-national Postpartum/non-postpartum	HH surveys
COVERAGE: care for women and girls with complications	Caesarean section rate^a^	Proportion of women and girls with a live birth delivered by caesarean section within a specified time period	Numbers deliveries by caesarean section	Total number of live births to women and girls in same period	Wealth quintile Residence Age Sub-national	HH surveys
INPUT: counting	Maternal death registration, including cause of death^a^	Proportion of maternal deaths registered with cause of death specified based on ICD-MM codes within a specified time period	Number of maternal deaths registered with cause of death specified based on ICD-MM codes	Total estimated number of maternal deaths in the same time period	Sub-national	Health facilities, CRVS, census
INPUT: Availability of care	Availability of functional EmOC facilities^a^ (per population)	At least five emergency obstetric care facilities (per 500,000 people) including at least one comprehensive and the rest basic emergency obstetric care facilities.	Number of obstetric care facilities that provided EmOC signal functions^d^ in the last three months	Per 500,000 population	Sub-national, Facility level	HF surveys, routine facility monitoring, census or other population data source

^a^ENAP indicator

^b^Link to WHO Quality of Care metrics

^c^Link to WHO 100 Core Indicators

^d^Signal functions; Basic: 1) parenteral antibiotics; 2) uterotonic drugs; 3) parenteral anticonvulsants for preeclampsia and eclampsia; 4) manual removal of placenta; 5) remove retained products (e.g. manual vacuum extraction, dilation and curettage); 6) perform assisted vaginal delivery (e.g. vacuum extraction, forceps delivery); and 7) basic neonatal resuscitation (e.g., with bag and mask); Comprehensive: All seven basic plus: 8) perform surgery (e.g., caesarean section); and 9) perform blood transfusion

The group was unanimous in its consensus to deliver this core set of maternal health indicators to WHO for further member state consultation and deliberation through global processes, including harmonization with core metrics from the ENAP and consideration as part of a combined monitoring framework for maternal and newborn health within the Indicator Framework of the Global Strategy for Women’s, Children’s and Adolescents’ Health (2016–2030).

Consensus was reached through extensive discussion of all proposed indicators (see Table 5). The final core list of indicators focuses primarily on routine care for all women; however, ending preventable maternal death requires appropriate management of maternal complications. The group discussed two potential composite indicators related to essential services for obstetric complications- Unmet Obstetric Need (UON: the proportion of women who receive surgery/major obstetric intervention among all women who experience a severe complication that is an indication for surgery/major obstetric intervention) and Met Need for Emergency Obstetric Care (the proportion of women who are expected to experience a complication who receive treatment) [8, 9]. Neither indicator met all the selection criteria for inclusion, due to issues related to feasibility and validity. For example, Met Need for EmOC assumes 15 % of women will experience a complication; this denominator has not been validated and may vary by setting. UON assumes 1 to 2 % of women will experience a severe complication, which has been validated in several settings. However the UON numerator is based on obstetric complications requiring surgical interventions which can be variable even for a single complication (e.g. pre-eclampsia/eclampsia (PEE) or postpartum hemorrhage (PPH) can often be managed without surgical intervention.) [9]. The group agreed that Met Need for EmOC was more appropriate for global monitoring, but that further refinement of the denominator would be needed for inclusion in a global monitoring framework. It was agreed that countries that are currently collecting Met Need for EmOC should continue to monitor this indicator at national and sub-national levels until the indicator has been updated.

**Table 5:** Maternal Health Indicators considered for global monitoring and reporting, by indicator selection criteria

Indicator	Criteria (√ = YES, ~ = Some, X = No)					Notes
	Relevance	Validity	Feasibility	Availability	Complements MH Monitoring FW^a^	
Impact						
Maternal mortality ratio	√	√	~	~	√	
Maternal cause of death	√	~	~	~	√	- Global Strategy indicator – need to strengthen information systems to routinely collect
*Case fatality rate*	√	~	~	~	X	- More feasible to collect at sub-national or service delivery levels
Adolescent birth rate	√	√	√	√	√	
COVERAGE: care for all women and girls						
4 or more antenatal care visits	√	√	√	√	√	
* Blood pressure screening during antenatal care*	√	√	√	√	X	- May focus ANC on only BP screening
* Full course of iron/folate during pregnancy*	√	X	√	√	X	- May focus ANC on only iron folate
Skilled attendant at birth	√	~	√	√	√	- SDG indicator
Institutional delivery	√	√	√	√	X	- Complements skilled attendant at birth
Oxytocin within 1 minute of birth (facility births)	√	~	~	~	X	- Indicates quality of delivery care - Feasible to strengthen routine health information systems to collect - Timing is challenging - Change wording to “uterotonic immediately after birth”
* Companion at birth*	√	~	X	X	X	- Respectful maternity care important, but this is only one element - May focus respectful maternity care only on companion at birth - Not feasible for many high-volume facilities - Not included in large-scale household surveys
Early postpartum care for woman	√	~	√	√	√	- Postnatal/postpartum care within 2 days of birth, regardless of place of delivery
Met Need for family planning	√	√	√	√	√	
COVERAGE: Care for women and girls with complications						
* Caesarean section rate among poor (bottom two quintiles)*	√	√	~	~	X	
Caesarean section rate	√	√	√	√	√	
* Met need for EmOC*	√	X	X	X	X	
* UnMet need for EmOC*	√	X	X	X	X	
INPUT: counting						
* Birth registration*	X	~	~	~	√	- Not relevant for maternal mortality – included in ENAP core metrics
Death registration, including cause of death	√	~	~	~	√	
INPUT: Availability of care						
Availability of EmOC per 500,000 population	√	~	~	~	X	- Essential to include an indicator on emergency obstetric care – indicator to be refined and updated

Indicators in italics NOT included in final core list

NOTE: √ = YES; ~ = Some; X = No

^a^SDGs, WHO 100 Core indicators, WHO Quality of Care metrics, Every Newborn Action Plan (ENAP)

There was widespread agreement on the need to refine the skilled birth attendant (SBA) indicator to increase its validity. WHO and UNICEF are working in this area to review and revise the current metadata and enhance measurement (personal communication, Chou and Amouzou), and the indicator met other inclusion criteria. Therefore, SBA was included in the core MH indicator list, with institutional delivery added as a supplemental indicator. There were also important discussions about how to interpret caesarean section rates, given the global ongoing debates about the appropriate population-based proportion and rising rates of voluntary caesarean sections. It was agreed that it will be particularly important for this indicator to be disaggregated by residence and socio-economic quintile, and increasingly to capture indications [10]. The group discussed a measure to indicate the availability of functional EmOC facilities. Some participants felt this indicator needed further refinement prior to inclusion, especially to re-define the denominator to be based on expected pregnancies/births instead of population. Ultimately, the group agreed that this indicator met the criteria for global monitoring and that the definition would be updated moving forward.

Particular areas of contention included whether measures of facility-level quality of care and coverage are useful and feasible for global monitoring and reporting (e.g. measures of specific content of antenatal, labor and delivery, and postpartum care). While many participants agreed that such measures are critically needed for program improvement and effectiveness at country level, there was a lack of consensus on whether or not quality measures should be included in a global monitoring framework as well as active debate about which indicator would adequately capture all elements of care. A single indicator for routine labor and delivery care—provision of uterotonic immediately after birth—was selected. In addition, the group strongly agreed on the importance of a high-level indicator of provision of respectful maternity care (RMC) and elimination of mistreatment in childbirth [11]. There was agreement that such metrics need further development and testing prior to inclusion for global monitoring and reporting. The development of indicators related to quality of antenatal care, postnatal care, RMC and Met Need for EmOC were flagged as an important priority for further development and testing. The group acknowledged ongoing work by WHO to define quality of care indicators for maternal and newborn care in health facilities as part of the WHO Quality of Care framework.

## Discussion

“What gets measured gets done.” This simple phrase captures the synergistic relationship between measurement and action [12], which is critical for achieving ambitious targets outlined in EPMM Strategy documents, the SDGs, and the UN Global Strategy for Women’s, Children’s, and Adolescent’s Health. This paper describes the results and process of defining core maternal health indicators for global monitoring and reporting as part of the EPMM strategy. Agreement on a core set of global maternal health indicators is important for galvanizing action and accountability to reduce preventable maternal mortality as well as to highlight technical areas needing further development and indicator testing. WHO will propose the agreed indicators for further member state consultation through global processes, including harmonization with Every Newborn Action Plan core metrics into a combined global monitoring framework for maternal and newborn health. All participants pledged their support for these processes.

The MDG5 goals and targets raised the profile of global maternal mortality reduction, galvanizing political will and resource allocation, which contributed to accelerated progress in mortality reduction. Between 1990 and 2000, MMR declined at an estimated average annual rate of 1.2 % globally, compared with an average of 3.0 % between 2005 and 2015 after the launch of the Millennium Development Goals in 2000 [1]. But accelerating further declines and ensuring equity will now require different strategies and concerted efforts to reach vulnerable and at-risk populations. A common global monitoring framework that includes key coverage and outcome indicators will be crucial to monitor whether strategies are working as intended to track progress toward global targets.

The core maternal health indicators are the result of a systematic sequential process to prioritize a small number of impact, coverage and input measures for global monitoring. These global indicators are not intended to address the measurement needs and priorities for improvement at all levels of the system across all contexts. Other complementary efforts are underway to address maternal and newborn health measurement needs related to processes and outcomes at national, subnational and service delivery levels. The core global level indicators complement these other measurement efforts and contribute to policy decision-making, resource mobilization, program planning, and aim to accountability for achieving preventable maternal deaths across the globe.

There has been promising progress in defining, testing and using indicators to monitor maternal health programs at subnational and service-delivery levels [13–15]. The global indicator on availability of functional EmOC facilities will build on this existing body of work to incorporate elements of basic maternity care, newborn care, human resources, travel time, and others. In addition, WHO is leading an initiative to define standards and metrics for quality of facility-based care around the time of birth. The WHO quality of care framework and associated metrics prioritize measures for use by managers and providers to drive improvements in maternal and newborn care at the point of service [16]. In collaboration with the ENAP metrics improvement plan, work is underway to validate several facility-based indicators, including the Phase I EPMM indicator uterotonic use immediately after birth. This validation work will include assessing whether the woman was aware that a uterotonic was given after birth to prevent postpartum hemorrhage.

There are also many efforts underway to develop and test innovative methods to generate higher quality data to more accurately track facility-level quality of care metrics routinely. Innovative approaches around combining population-based surveys and health facility surveys are in progress [17, 18] as well as unique methods for routine monitoring of signal functions for obstetric and newborn care through quarterly monitoring visits (personal communication, UNFPA). Furthermore, there are efforts underway to use geographic information systems (GIS) to capture access to care and visualize data at sub-national levels to improve decision making [19]. As these methods are tested and refined, they will greatly contribute to more robust and accurate data to track program implementation and outcomes, which can be utilized at sub-national and service delivery levels to improve quality of care, and ultimately feed into global level monitoring and reporting. To allow for evolving improvements in measurement strategies and data quality, the global monitoring framework must be dynamic and flexible to incorporate new developments and indicators.

There were some limitations. First, because the goal was to reach expert consensus on technically sound indicators for monitoring progress toward global maternal health goals, the timeframe was dictated by time-bound global political processes, including the launch of the SDGs and the Global Strategy. As a result, it was not possible to conduct a lengthy process utilized in other indicator prioritization exercises or research studies. However, every attempt was made to follow a systematic and transparent process, including a series of virtual consultations with experts from diverse backgrounds based on pre-determined principles, indicator selection criteria, and standard questions, which culminated with an in-person meeting. This process provided a transparent mechanism for consensus-building, within the time constraints. In total, over 45 stakeholders participated in the process; however, there were limited participants from the country level. WHO will conduct further consultations with member states through global processes, and the core MH indicators from this process have been introduced into consultations regarding indicator selection for the UN Global Strategy for Women’s, Children’s and Adolescent’s Health.

Second, to meet the aim of a core set of indicators acceptable for global monitoring and reporting by all countries, the issues of feasibility, such as current data availability and quality, were by necessity balanced against the desire to “push the envelope” and drive accelerated health information system improvements. The group attempted to deal with this tension openly in the discussions and some indicators that will require further refinement and testing were included for this reason while others were not. For example, maternal cause of death is included as a core indicator, although further work is needed to standardize diagnosis, coding, and promote recording of all deaths.

Finally, monitoring the core set of indicators for maternal health cannot be implemented without attention to data systems and efforts to ensure adequate data quality. We must support countries in developing and implementing plans to routinely collect these indicators through existing data systems and surveys, such as national health management information systems, household survey programs such as Demographic and Health Surveys (DHS) or Multiple Indicator Cluster Surveys (MICS), and health facility assessments such as the Service Availability and Readiness Assessment (SARA), the Service Provision Assessment (SPA), and not create parallel structures. The recently launched Health Data Collaborative is working to coordinate investments and build capacity at country level to collect indicators required for SDG monitoring.

The global *Strategies toward Ending Preventable Maternal Mortality* [2] highlights an overall call to improve metrics, measurement systems and data quality. Representing one among several essential components of that goal, a core set of global indicators is key to galvanizing action and prioritizing attention to maternal health and survival, driving investment in the improvement of services, delivery systems and other proximal determinants, and providing a mechanism for accountability to stakeholders. The next phase of this process aims to develop consensus on measures to address the more distal causes of maternal survival and health, drawing from the key objectives in EPMM strategic framework that focus on the underlying social, political, and economic determinants of maternal health and survival, providing a pathway for countries to operationalize the EPMM strategic objectives and measure change over time.

The SDGs are intended to realize the rights of all and to achieve gender equality and the empowerment of women and girls, balancing the dimensions of sustainable development: the economic, social, and environmental [20]. The UN Secretary General’s Global Strategy for Women’s, Children’s, Adolescent’s Health addresses health across sectors and SDGs to move beyond the focus on survival to thriving and transformation. The Indicator and Monitoring Framework for the Global Strategy describes 16 key indicators considered the *minimal* subset of a total of 60 indicators to provide assessment of progress towards the implementation of the Global Strategy objectives [21]. The EPMM Phase I indicators maternal mortality ratio and adolescent birth rate are included within this minimum dataset, however expanded monitoring of outcomes related to the key themes of EPMM will help to fully realize and address priority issues in the SDGs, including equity, human resources, gender, and human rights. Taken together, the development of the EPMM Phase I and II indicators will track progress and address a comprehensive set of issues related to not only “survival” but also “thrive and transform”; together they track progress.

## Conclusion

Within the global maternal health community, there has been a lack of consensus on core metrics for monitoring maternal health status, health system performance and program implementation necessary to achieve EPMM targets. This is largely due to the complex nature of maternal health programs that must address a wide range of health determinants and deliver a package of services across a continuum within the context of differing political priorities, health system structures, and in coordination with non-health sector actors. However, the lack of robust measures, functioning measurement systems, and data of reliable quality have stymied efforts to accurately track whether countries are meeting global health targets. It is important that members of the global and maternal health communities work together through participatory processes to track progress at all levels (global, regional, national, subnational, and facility) to end preventable maternal mortality. This paper describes a core set of maternal health indicators for global monitoring and reporting for all countries, developed through an inclusive, iterative, and consultative process led by WHO on behalf of the EPMM Working Group. Monitoring these core indicators will be critical to support achievement of the global targets outlined in the SDGs and ultimately to end preventable maternal mortality.

## Additional files

Additional file 1: Summary of feedback from stakeholders on proposed EPMM Phase 1 indicators. (XLSX 69 kb) Additional file 2: Core Maternal Health Indicators for Global Monitoring and Reporting and linkages to Every Newborn Action Plan (ENAP) indicators. (DOCX 23 kb)

## Abbreviations

AMDD: Averting maternal death and disability program
EmOC: Emergency obstetric care
ENAP: Every newborn action plan
EPMM: Ending preventable maternal mortality
MCSP: Maternal and child survival program
MDG: Millennium development goal
MH: Maternal health
MHTF: Maternal health task force
MMR: Maternal mortality ratio
PEE: Pre-eclampsia/eclampsia
PPH: Postpartum hemorrhage
RMC: Respectful maternity care
SBA: Skilled birth attendant
SDG: Sustainable development goals
USAID: United States agency for international development
WHO: World health organization
