# Supplementary material for: A common monitoring framework for ending preventable maternal mortality, 2015–2030: phase I of a multi-step process
Source: BMC Pregnancy Childbirth. 2016 Aug 26;16(1):250. doi: 10.1186/s12884-016-1035-4 (PMC5002107; doi:10.1186/s12884-016-1035-4)
Supplement: Additional file 2: — Core Maternal Health Indicators for Global Monitoring and Reporting and linkages to Every Newborn Action Plan (ENAP) indicators. (DOCX 23 kb) [file 12884_2016_1035_MOESM2_ESM.docx]

Additional file 2: Core Maternal Health Indicators for Global Monitoring and Reporting and linkages to Every Newborn Action Plan (ENAP) indicators

| **IMPACT** | **EPMM Indicators** | **ENAP Indicators** |
| --- | --- | --- |
|  | **1. Maternal mortality ratio ^** | **1. Maternal mortality ratio** |
|  | 2. Maternal cause of death  (direct/indirect) based on ICD-MM |  |
|  | 3. Adolescent birth rate ^ |  |
|  |  | 2. Stillbirth rate  *Additional Indicator*: Intrapartum stillbirth rate |
|  |  | 3.Neonatal mortality rate  *Additional Indicators*:   - Preterm birth rate - Small for gestational age - Neonatal morbidity rates - Disability after neonatal conditions |
| **COVERAGE: care for all women** | 4. Four or more antenatal care visits ^  ***Priority for Indicator Development*: Content of antenatal care** | ***Additional Indicator:* Content of antenatal care** |
|  | **5.Skilled attendant at birth ^** | **4.Skilled attendant at birth** |
|  | 6.Institutional Delivery  *Priority for Indicator Development*:  Respectful maternity care |  |
|  | **7. Early postnatal/postpartum care for woman and baby (within 2 days of birth) ^**  *Priority for Indicator Development:* Content of postnatal care | **5.Early postnatal care for mothers and babies** |
|  | 8. Met need for family planning ^ |  |
|  | 9. Uterotonic immediately after birth for prevention of post-partum hemorrhage (among facility births) ** |  |
|  |  | 6.Essential newborn care  (tracer is early breastfeeding)  *Additional Indicator*:  Exclusive breastfeeding up to 6 months |
| **COVERAGE: care for women and newborns with complications** | **EPMM Indicators** | **ENAP Indicators** |
|  |  | 7.Antenatal corticosteroid use |
|  |  | 8.Newborn resuscitation |
|  |  | 9.Kangaroo mother care, feeding support |
|  |  | 10.Treatment of severe neonatal sepsis  *Additional Indicator:*  Chlorhexidine cord cleansing |
|  | **10. Caesarean section rate**  *Priority for Indicator Development:* Met need for Emergency Obstetric Care | ***Additional indicator*:**  **Caesarean section rate** |
| **INPUTS: Counting** | 11. Maternal death registration | Birth registration  *Additional indicator:*   - Death Registration, cause of death |
| **INPUT: Availability of care** | 12. **Availability of functional Emergency Obstetric Care facilities** | - **Emergency Obstetric Care** - Care of small and sick newborns - Every Mother Every Newborn Quality Initiative with measurable norms and standards |

** Link to WHO Quality of Care metrics , ^ Link to WHO 100 Core indicators,
